# Comparative Analysis of Binary Similarity Measures for Compound Identification in Mass Spectrometry-Based Metabolomics

**DOI:** 10.3390/metabo12080694

**Published:** 2022-07-26

**Authors:** Seongho Kim, Ikuko Kato, Xiang Zhang

**Affiliations:** 1 Biostatistics and Bioinformatics Core, Karmanos Cancer Institute, Department of Oncology, School of Medicine, Wayne State University, Detroit, MI 48201, USA; 2 Department of Oncology and Pathology, School of Medicine, Wayne State University, Detroit, MI 48201, USA; katoi@karmanos.org; 3 Department of Chemistry, University of Louisville, Louisville, KY 40292, USA; xiang.zhang@louisville.edu

**Keywords:** binary similarity measure, compound identification, EI, ESI, mass spectrometry, untargeted metabolomics

## Abstract

Compound identification is a critical step in untargeted metabolomics. Its most important procedure is to calculate the similarity between experimental mass spectra and either predicted mass spectra or mass spectra in a mass spectral library. Unlike the continuous similarity measures, there is no study to assess the performance of binary similarity measures in compound identification, even though the well-known Jaccard similarity measure has been widely used without proper evaluation. The objective of this study is thus to evaluate the performance of binary similarity measures for compound identification in untargeted metabolomics. Fifteen binary similarity measures, including the well-known Jaccard, Dice, Sokal–Sneath, Cosine, and Simpson measures, were selected to assess their performance in compound identification. using both electron ionization (EI) and electrospray ionization (ESI) mass spectra. Our theoretical evaluations show that the accuracy of the compound identification was exactly the same between the Jaccard, Dice, 3W-Jaccard, Sokal–Sneath, and Kulczynski measures, between the Cosine and Hellinger measures, and between the McConnaughey and Driver–Kroeber measures, which were practically confirmed using mass spectra libraries. From the mass spectrum-based evaluation, we observed that the best performing similarity measures were the McConnaughey and Driver–Kroeber measures for EI mass spectra and the Cosine and Hellinger measures for ESI mass spectra. The most robust similarity measure was the Fager–McGowan measure, the second-best performing similarity measure in both EI and ESI mass spectra.

## 1. Introduction

Compound identification is a critical, but challenging and time-consuming, task in untargeted metabolomics [1,2]. In general, there are two approaches widely implemented for compound identification, which are structure-based and mass spectral library-based methods. The mass spectral library-based approach is to compare a query mass spectrum against all of the mass spectra in a mass spectral library, followed by ranking the library spectra according to their similarity scores; selecting the compound in the mass spectral library that has the highest similarity score compared to the compound that has given rise to the query spectrum. On the other hand, given a query mass spectrum, the structure-based approach first predicts the mass spectrum of a compound, using its chemical structure information. It then compares the query spectrum to all of the simulated mass spectra, followed by ranking them based on their similarity scores and selecting the compound with the highest similarity score compared to the identification result.

The most important component of both of the approaches is the similarity measure. Two types of similarity measures were used: continuous and binary similarity measures. The continuous similarity measures calculate the similarity scores based on the continuous intensity values. In contrast, the binary similarity measures compute the scores based on the binary strings representing the presence or absence of nonzero intensities [3]. The continuous similarity measures, including the composite measures of continuous and binary similarity measures, are utilized essentially for the mass spectral library-based approach, and the structure-based approach only adapts the binary similarity measures. The continuous intensity values must be available for the continuous similarity measures, but the current technologies cannot yet simulate reliable continuous intensity values [1,4,5,6]. This is why binary similarity measures are the only choice of structure-based approaches.

Various continuous similarity measures have been developed, primarily for gas chromatography–mass spectrometry (GC-MS)-based metabolomics, such as dot product [7], probability-based matching system [8], the Hertz similarity index [9], absolute value distance (L1-nrom) [10], normalized Euclidean distance (L2-norm) [11], the Fourier and Wavelet transform-based composite measures [12], and semi-partial and partial correlation-based measures [13]. For liquid chromatography–mass spectrometry (LC-MS)-based metabolomics, the development of the continuous similarity measures has begun to receive attention relatively recently, and a few new measures have been developed, including Spec2Vec [14], and spectral entropy similarity measures [15]. Nevertheless, the most widely used continuous similarity measure is still the famous dot product.

On the other hand, many binary similarity measures have been introduced for areas such as psychology, ecology, and chemoinformatics, not for compound identification [3]. Numerous studies surveyed and compiled binary similarity measures [3,16,17,18,19,20,21,22,23,24,25]. For instance, Choi et al. [18] rendered a comprehensive survey, using 76 binary similarity and distance measures, which, to our knowledge, was the most comprehensive collection. Todeschini et al. [3] did a comparative analysis of 51 different binary similarity measures in terms of the theoretical characteristics and the effectiveness of virtual screening. Brusco et al. [17] compared 71 binary similarity measures for cluster analyses in psychological applications. Notwithstanding, no-one has carried out a comparative study to evaluate their performances in compound identification. The Jaccard [26], also known as the Tanimoto, similarity measure has been widely used in the structure-based compound identification without methodological justification. Therefore, there is an urgent need for a comprehensive study in terms of compound identification.

Thus, we aimed to evaluate the performances of the binary similarity measures in compound identification, and produce general guidance when a binary similarity measure is used in untargeted metabolomics. To this end, as described in Section 4, in compound identification, two compounds will be considered similar if both have many common m/z values with nonzero intensity, so we mainly focused on asymmetric measures. This led us to select 15 binary similarity measures, including the well-known Jaccard, Dice, Sokal–Sneath, Cosine, and Simpson measures, that could be utilized for the compound identification, based on the list of similarity and distance measures collected by Choi et al. [18]. The performances in compound identification were evaluated in two ways. One was from a theoretical perspective, and the other was from a practical perspective, using both electron ionization (EI) and electrospray ionization (ESI) mass spectral libraries for GC-MS-based and LC-MS-based untargeted metabolomics, respectively. It should be noted that no continuous similarity measures were considered because the purpose of this study is to evaluate only binary similarity measures.

## 2. Results

We evaluated 15 binary similarity measures in terms of compound identification. As described in Table 4 of Section 4, the selected 15 binary measures are Jaccard (1), Dice (2), 3W-Jaccard (3), Sokal–Sneath (4), Cosine (5), Mountford (6), McConnaughey (7), Driver–Kroeber (8), Simpson (9), Braun–Banquet (10), Fager–McGowan (11), Kulczynski (12), Intersection (13), Hamming (14), and Hellinger (15). Note that the numbers in parentheses represent the indices of the selected binary measures, as shown in Table 4 of Section 4, and we will use the indices and the names interchangeably. First, the relationships among all of the binary similarity measures were assessed theoretically. Then, their performances in compound identification were compared, using the EI and ESI mass spectra. In particular, because compound identification in this study is based on binary mass spectra, the EI and ESI mass spectra were first converted to binary strings with non-zero intensity to mimic the predicted binary mass spectra in structure-based compound identification. We then evaluated the performance of the binary measurements.

### 2.1. Theoretical Considerations

We begin with a definition of the strictly order-preserving similarity measures. Let $Q=\{q_{i}|1\leq i\leq n\}$ and $R=\{r_{i}|1\leq i\leq m\}$ be the set of query binary mass spectra and the reference binary mass spectra, respectively, where n=|Q| and m=|R|, and let S(q,r) be a binary similarity measure between the query and reference binary mass spectra. Note that the reference binary mass spectra could be constructed through prediction in the structure-based compound identification.

**Definition** **1.** *Two binary similarity measures*$S_{1}$*and*$S_{2}$*are strictly order-preserving if and only if, for all*q∈Q*,*$S_{1}\left(q,r_{i}\right)<S_{1}\left(q,r_{j}\right)$ $\overset{}{\Leftrightarrow }$ $S_{2}\left(q,r_{i}\right)<S_{2}\left(q,r_{j}\right)$*for any*$r_{i},r_{j}\in R$*with*i≠j*.*

Both mass spectral library-based and structure-based approaches calculate the similarity scores between a query and all of the reference mass spectra and rank the scores from largest to smallest. The reference compound with the largest score will then be matched to the query mass spectrum. In other words, compound identification depends on the ranks, not the magnitude of the spectrum similarity scores. Therefore, if the similarity score order between the two binary similarity measures is maintained, the identification accuracy is the same, which can be restated as follows:

**Theorem** **1.** *If two binary similarity measures* $S_{1}$*and*$S_{2}$*are strictly order-preserving, then*$A\left(S_{1}\right)=A\left(S_{2}\right)$*, where*A(S)*is the accuracy of the similarity measure*S*.*

Therefore, any binary similarity measures that preserve the orders strictly will have identical identification accuracy. Accordingly, we assessed the mathematical expressions of the 15 binary similarity measures, as available in Table 4 of Section 4, and obtained the following relationships:

(1) The similarity measures 1 (Jaccard), 2 (Dice), 3 (3W-Jaccard), 4 (Sokal–Sneath), and 12 (Kulczynski) are strictly order preserving;
(2) The similarity measures 5 (Cosine) and 15 (Hellinger) are strictly order preserving;
(3) The similarity measures 7 (McConnaughey) and 8 (Driver–Kroeber) are strictly order preserving.

Consequently, by Theorem 2.1, the similarity measures 1 (Jaccard), 2 (Dice), 3 (3W-Jaccard), 4 (Sokal–Sneath), and 12 (Kulczynski) have the identical accuracy, and so do 5 (Cosine) and 15 (Hellinger), as well as 7 (McConnaughey) and 8 (Driver–Kroeber). Note that it has been known that measures 7 (McConnaughey) and 8 (Driver–Kroeber) have the perfect correlation, as shown in Todeschini et al. [3] and Brusco et al. [17]. Thus, it is obvious that their similarity measures are strictly order preserving. For the other two relationships, the detailed proofs are available in the Supplementary Materials. We will denote a group of binary similarity measures that have identical accuracy by {⋅} and, for instance, {5,15} indicates that the binary similarity measures 5 and 15 have the same accuracy.

### 2.2. EI Mass Spectra-Based Identification

We compared the identification performance of the binary similarity measures, using EI mass spectra. The characteristics of the similarity measures were investigated in terms of the densities and the correlations of the similarity scores of the binary similarity measures. Furthermore, the accuracy of the compound identification was computed, according to the set of compounds with higher similarity scores.

#### 2.2.1. Scores of Binary Similarity Measures

**Figure 1:**
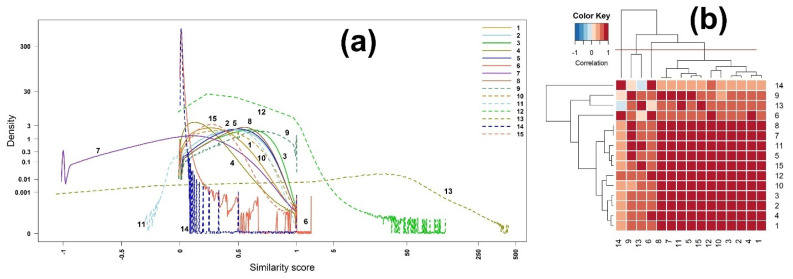
(**a**) Densities and (**b**) heatmap of the correlation matrix of scores among 15 binary similarity measures for EI mass spectra-based compound identification. The correlation was calculated using Pearson’s correlation coefficients. The horizontal red solid line indicates the four clusters generated by hierarchical clustering. The numbers in row and column represent the indices of binary similarity measures corresponding to Table 4 of Section 4.

The densities of the scores of similarity measures are plotted in Figure 1a. The density is the probability density function (pdf) of the scores that was empirically computed, using kernel density estimation. As expected, the maximum ranges of the scores of the measures 6 (Mountford), 12 (Kulczynski), and 13 (Intersection) are greater than 1, while the minimum ranges of the measures 7 (McConnaughey) and 11 (Fager–McGowan) are less than 0.

The correlation matrix and its heatmap of the scores are presented in Table S1 (Supplementary Materials) and Figure 1, respectively. We further grouped the 15 binary similarity measures into four clusters, using hierarchical clustering, resulting in the following four groups: (14); (9, 13); (6); and (8, 7, 11, 5, 15, 12, 10, 3, 2, 1), as shown in Figure 1b. Interestingly, the correlation coefficient between the measures 7 (McConnaughey) and 8 (Driver–Kroeber) is one (Table S1, Supplementary Materials), while the correlation between the measures 13 (Interaction) and 14 (Hamming) is negative, indicating that the correlation has nothing to do with the density shape.

#### 2.2.2. Accuracies of Binary Similarity Measures

**Figure 2:**
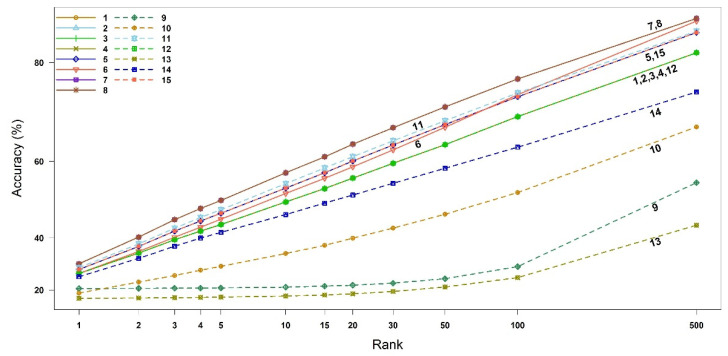
Accuracy of all similarity measures for EI mass spectra-based compound identification by rank. The *x*-axis represents the ranks and the *y*-axis the identification accuracy. The numbers in legend and plot are the indices of binary similarity measures corresponding to Table 4 of Section 4.

We calculated the compound identification accuracy and its associated 95% confidence interval (CI), up to the top three highest similarity scores (i.e., Rank 1, 2, and 3). The 95% CIs of the accuracies were obtained using bootstrap resampling methods with 10,000 replicates, as shown in Table 1. As derived theoretically in Section 2.1, the binary similarity measures {1, 2, 3, 4, 12}, measures {5, 15} and measures {7, 8} indeed have the same accuracies. The measures 7 (McConnaughey) and 8 (Driver–Kroeber) outperform the other measures in all three ranks, followed by measure 11 (Fager–McGowan). The worst measure of similarity is 13 (Inter-section). The 95% CI of measure 11 overlaps with those of the measures {5, 15}. Interestingly, measure 10 becomes better than measure 9 from Rank 2. The accuracies and 95% CIs up to Rank 500 are available in Figure 2 and Table S2 (Supplementary Materials).

**Figure 3:**
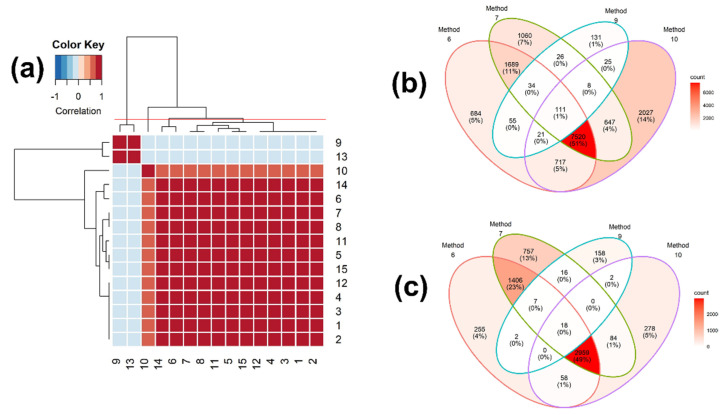
(**a**) Heatmap of the correlation matrix of the identification results among 15 binary similarity measures for Rank 1 and (**b**,**c**) Venn diagrams of consensus analysis among 4 selected binary similarity measures for EI mass spectra-based compound identification. In (**a**), the correlation was calculated using Pearson’s correlation coefficients. The horizontal red solid line indicates the four clusters generated by hierarchical clustering. The numbers in row and column represent the indices of binary similarity measures corresponding to Table 4 of Section 4. In (**b**), the Venn diagram was constructed based on all reference compounds with the highest corresponding similarity scores. In (**c**), the Venn diagram was constructed based on all reference compounds that were corrected identified.

The hierarchical clustering among the 15 measures was carried out using the identification results with the highest similarity scores, i.e., Rank 1. As shown in Figure 3a and Table S3 (Supplementary Materials), the correlation coefficients between the two measures, 9 (Simpson) and 13 (Intersection), and the other measures are all negative. We also grouped the 15 binary similarity measures into four clusters, using hierarchical clustering, resulting in (9, 13), (10), (6, 14), and ({1, 2, 3, 4, 12}, {5, 15}, {7, 8}, 11), which is different from those for the scores of the similarity measures in Figure 1. The hierarchical clustering and heatmaps up to Rank 500 are depicted in Figure S1 (Supplementary Materials). The group memberships of the hierarchical clustering are slightly changed at Rank 10, 50, and 500.

We further selected the similarity measures with the highest accuracy from each cluster, which are measures 6, 7, 9, and 10, and then carried out the consensus analysis of these four similarity measures, using all of the reference compounds that were matched to the query mass spectra (Figure 3b) and all of the reference compounds that were correctly matched (Figure 3c). Of these, measure 7 has the highest accuracy, followed by measures 6 and 9. Measure 6 has the lowest accuracy, but detected the most unique compounds (14%; Figure 3b). As expected, measure 7 identified the most unique true compounds (13%; Figure 3c). Measure 9 detected the least unique compounds (1%; Figure 3b) and true compounds (3%; Figure 3c).

### 2.3. ESI Mass Spectra-Based Identification

Using the ESI mass spectra data, the identification performance of the binary similarity measures was evaluated. Similar to the EI mass spectra-based identification, the densities and correlations of the similarity scores, and the accuracies of the compound identification of the binary similarity measures were investigated.

#### 2.3.1. Scores of Binary Similarity Measures

**Figure 4:**
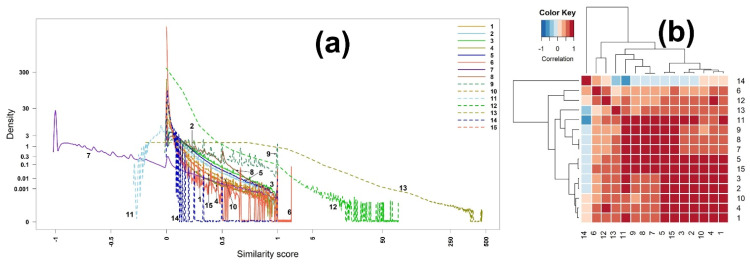
(**a**) Densities and (**b**) heatmap of the correlation matrix of scores among all 15 binary similarity measures for ESI mass spectra-based compound identification. The correlation was calculated using Pearson’s correlation coefficients. The horizontal red solid line indicates the four clusters generated by hierarchical clustering. The numbers in row and column represent the indices of binary similarity measures corresponding to Table 4 of Section 4.

Figure 4a depicts the densities of the scores of similarity measures. Compared to the densities of EI mass spectra-based identification (Figure 1a), the ranges of scores are similar to each other, except for measure 13 (Intersection) which has a narrower range than that of the EI identification. Still, the shapes are considerably different from each other.

The heatmap of the scores is slightly different from that of the EI mass spectra-based identification, as shown in Figure 4b. In particular, measure 14 (Hamming) is negatively correlated with measures 13, 11, 9, 8, 7, 5, 15, 3, and 2, while it was positively associated with them in the case of the EI mass spectra-based identification. The four groups clustered by hierarchical clustering are (14), (6, 12), (13, 11, 9, 8, 7), and (5, 15, 3, 2, 10, 4, 1), which is slightly different from those of the EI mass spectra-based identification. Similar to what was observed in Table S1 (Supplementary Materials), the correlation coefficient between measures 7 (McConnaughey) and 8 (Driver–Kroeber) is one (Table S4, Supplementary Materials).

#### 2.3.2. Accuracies of Binary Similarity Measures

**Figure 5:**
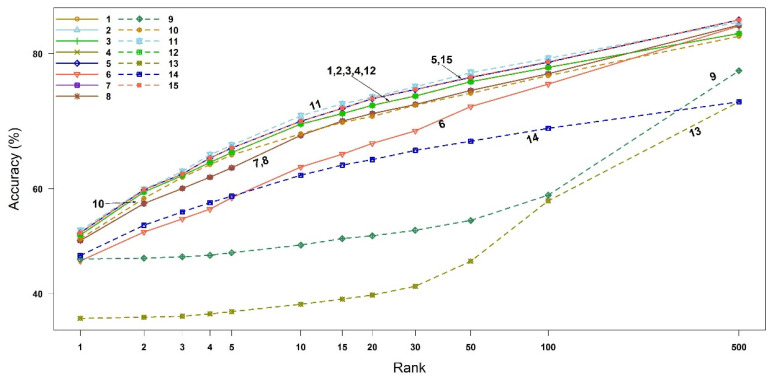
Accuracy of all similarity measures for ESI mass spectra-based compound identification by rank. The *x*-axis represents the ranks and the *y*-axis the identification accuracy. The numbers in legend and plot are the indices of binary similarity measures corresponding to Table 4 of Section 4.

The compound identification accuracy and its associated 95% CI up to Rank 3 are available in Table 2, where the 95% CIs were estimated using bootstrap resampling methods with 10,000 replicates. As expected, similar to the EI mass spectra-based identification, we can again confirm the theoretical facts in Section 2.1. that the binary similarity measures {1, 2, 3, 4, 12}, measures {5, 15} and measures {7, 8} have the same accuracies. Unlike the EI mass spectra-based identification, measures 5 (Cosine) and 15 (Hellinger) perform better than the other measures in all three ranks, followed by measure 11 (Fager–McGowan). The worst measure of similarity is 13 (Intersection), which is consistent with the EI mass spectra-based identification. The 95% CIs of the measures {1, 2, 3, 4, 12}, {5, 15}, {7, 8}, 10, and 11 overlap. The accuracies and 95% CIs up to Rank 500 are available in Figure 5 and Table S5 (Supplementary Materials). Interestingly, measures 6 (Mountford) and 9 (Simpson) become better than measure 14 (Hamming) from Rank 10 and 500, respectively. Overall, the identification accuracies are much higher than those of the EI mass spectra-based identification.

**Figure 6:**
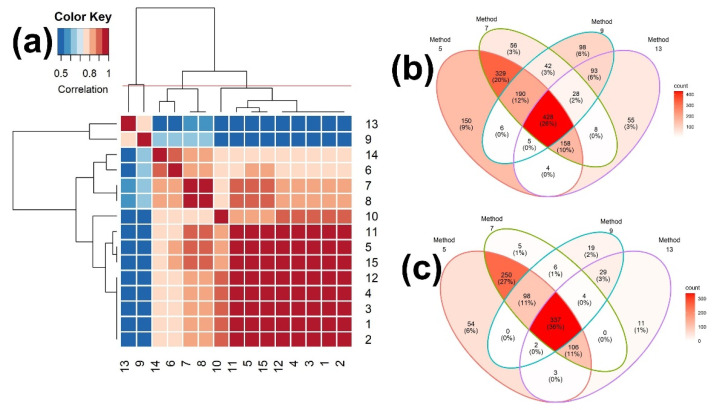
(**a**) Heatmap of the correlation matrix of identification among all 15 binary similarity measures for Rank 1 and (**b**,**c**) Venn diagrams of consensus analysis among 4 selected binary similarity measures for ESI mass spectra-based compound identification. In (**a**), the correlation was calculated using Pearson’s correlation coefficients. The horizontal red solid line indicates the four clusters generated by hierarchical clustering. The numbers in row and column represent the indices of binary similarity measures corresponding to Table 4 of Section 4; In (**b**), the Venn diagram was constructed based on all reference compounds with the highest corresponding similarity scores; In (**c**), the Venn diagram was constructed based on all reference compounds that were corrected identified.

Using the identification results with Rank 1, the hierarchical clustering was performed and depicted in Figure 6a and Table S6 (Supplementary Materials). As with the EI mass spectra-based identification, the associations between the two measures, 9 (Simpson) and 13 (Intersection), and the other measures are all negative. Based on the hierarchical clustering, the 15 binary similarity measures were grouped into four clusters, resulting in (13), (9), (14, 6, {7,8}), and (10, 11, {5, 15},{1, 2, 3, 4, 12}), which is different from those for the scores of similarity measures in Figure 4b and from those for the EI mass spectra-based identification in Figure 3a. Figure S2 (Supplementary Materials) depicts the hierarchical clustering and heatmaps up to Rank 500. At Ranks 5, 10, 30, and 100, the group memberships of hierarchical clustering are slightly changed.

The binary similarity measures with the highest accuracy from each of the four clusters, which are measures 5 (Cosine), 7 (McConnaughey), 9 (Simpson), and 13 (Intersection), were chosen. Then, the consensus analyses of these four similarity measures were performed, using all of the reference compounds that were matched to query mass spectra (Figure 6b) and all of the reference compounds that were correctly matched (Figure 6c). Of these, measure 5 has the highest accuracy, followed by measures 7 and 9. Measure 13 has the lowest accuracy and detected the least unique compounds (3%; Figure 6b). As expected, measure 5 detected the most unique compounds (9%; Figure 6b) and the most unique true compounds (6%; Figure 6c). Measure 7 detected the least unique true compounds (1%; Figure 6c).

## 3. Discussion

Using both the EI and ESI mass spectra, we compared the distributions of 15 binary asymmetric similarity measures in terms of their associations of similarity scores and their performances on compound identification. To our knowledge, this is the first comprehensive comparative study of binary similarity measures on compound identification.

The characteristics of the binary similarity measures are generally consistent between the similarity scores and identification outcomes, but for some of the similarity measures, this consistency is not preserved. For instance, although measures 9 (Simpson) and 13 (Intersection) have the positive correlations with other measures, except for measure 14 (Hamming), in terms of similarity scores (Figure 1b and Figure 4b), their identification-based correlation coefficients are negative with all of the other measures (Figure 3a and Figure 6a).

The best performing similarity measures are 7 (McConnaughey) and 8 (Driver–Kroeber) for EI mass spectra-based identification (Table 1), while measures 5 (Cosine) and 15 (Hellinger) have the highest accuracy for ESI mass spectra-based identification (Table 2). This confirms that the identification relies on the type of mass spectrum and is generally data-dependent, as seen in the previous study [27]. Moreover, regardless of the type of mass spectrum, measure 11 (Fager–McGowan) is the second-best performing similarity measure, signifying that measure 11 is data-independent and the most robust binary similarity measure, so it can be the choice when the data quality is not clear. It is worth noting that, as shown in Section 2.1, measures 7 and 8 have an identical performance in compound identification with a similarity score-based correlation coefficient of one, and measures 5 and 15 also have an identical identification performance, with the large similarity score-based correlation.

The identification accuracies of the similarity measure 14 (Hamming) are higher than those of the similarity measure 13 (Intersection), regardless of the type of mass spectrum (Table 1 and Table 2). Measure 13 uses only ‘c’, which is the number of m/z values with nonzero intensities present in both the query and target mass spectra (see Table 3 and Table 4). On the other hand, measure 14 uses only ‘a’ and ‘b’, which are the number of m/z values with nonzero intensities present in the query mass spectra only, and target mass spectra only, respectively. This implies that the mutually exclusive information, ‘a’ and ‘b’ plays a more critical role than the common information, ‘c’, in distinguishing the characteristics of the mass spectra.

In terms of mathematical expression (Table 4), all of the best performing measures 5, 7, and 11 have the term ‘(a+c)·(b+c)’ commonly in their denominators, and the expression of measure 5 and its square are one of the terms in the expressions of the measures 11 and 7, respectively. Indeed, these characteristics make the score-based correlation coefficients large among these three measures (Tables S1 and S4, Supplementary Materials). Particularly, the term ‘c^2^/{(a+c)·(b+c)}’ is the product of ‘c/(a+c)’ and ‘c/(b+c),’ which can be interpreted as the product of the proportions of the common information, ‘c’, among the m/z values with nonzero intensities for query and library mass spectra, respectively (Table 3).

In our study, ESI mass spectra-based identification exhibits a higher accuracy than EI mass spectra-based identification. This might be caused by the distributions of the similarity scores. That is, the similarity scores for the ESI mass spectra are more highly positively skewed than those for the EI mass spectra, resulting in a better discrimination ability. On the other hand, the EI mass spectra has nominal mass resolution, while the ESI mass spectrum has a much-improved resolution. The high resolution mass spectrum greatly increases the accuracy of calculating the a, b, and c terms in the binary measures.

Despite the fact that the Jaccard measure is commonly used for the structure-based identification, our study shows that the Jaccard measure is the third best performing measure in both the EI and ESI mass spectra-based identification. Moreover, the Jaccard measure has an identical identification accuracy with the Dice, 3W-Jaccard, Sokal–Sneath, and Kulczynski measures, although their mathematical expressions are different.

## 4. Materials and Methods

### 4.1. Binary Similarity Measures

Consider two mass spectra: one is a query spectrum $X=\left(x_{1},x_{2},\ldots ,x_{n}\right)$ and a reference spectrum $Y=\left(y_{1},y_{2},\ldots ,y_{n}\right)$, where $0\leq x_{i}<\infty$ and $0\leq y_{i}<\infty$ are the intensities of the ith mass to charge (m/z) value (i.e., fragment ion value) in X and Y, respectively, and n is the total number of m/z values. To calculate a binary similarity score, the first step is to convert each intensity into binary with 1 if the intensity is nonzero and 0 otherwise, using the function b(x)=0 if x=0;1 if x=1. Accordingly, the resulted binary query and reference mass spectra will be $Q=b\left(X\right)=\left(b\left(x_{1}\right),b\left(x_{2}\right),\ldots ,b\left(x_{n}\right)\right)=\left(q_{1},q_{2},\ldots ,q_{n}\right)$ and $R=b\left(Y\right)=\left(b\left(y_{1}\right),b\left(y_{2}\right),\ldots ,b\left(y_{n}\right)\right)=\left(r_{1},r_{2},\ldots ,r_{n}\right)$, respectively, where $q_{i},r_{i}\in \left\{0,1\right\},\;i=1,\ldots ,n$. The next step is to generate a confusion matrix between binary query and reference mass spectra, as shown in Table 3.

The confusion matrix summarizes the occurrences of the possible matches of binary intensities between the query and reference mass spectra. ‘a’, ‘b’, ‘c’, and ‘d’ denote the numbers of m/z values with nonzero intensity for query and zero intensity for reference (i.e., $q_{i}=1,\;r_{i}=0$), with zero intensity for query and nonzero intensity for reference (i.e., $q_{i}=0,\;r_{i}=1$), with nonzero intensities for both query and reference (i.e., $q_{i}=r_{i}=1$), and with zero intensities for both query and reference (i.e., $q_{i}=r_{i}=0$), respectively. Note that a+b+c+d=n. The binary similarity measures calculate the scores using ‘a’, ‘b’, ‘c’, and/or ‘d’. In particular, Todeschini et al. grouped the binary similarity measures according to the use of ‘d’ [3]. That is, if ‘d’ is used, it is called a symmetric similarity measure, otherwise, it is called an asymmetric similarity measure. In the mass spectrum-based compound identification, the asymmetric measures are broadly used because two compounds can be considered similar if both have many common m/z values with nonzero intensity [28,29]. Therefore, we focused solely on asymmetric similarity measures in this study and collected 15 literature-reported binary measures, as shown in Table 4 [3,18]. All of the similarity measures have a range of 0 to 1, except for the measures 6 (Mountford), 7 (McConnaughey), 11 (Fager–McGowan), 12 (Kulczynski), and 13 (Intersection), which have ranges of [0, 2], [−1, 1), (−1/2, 1), [0, ∞), and [0, ∞), respectively.

### 4.2. Mass Spectra Libraries

We considered two types of mass spectra libraries: electron ionization (EI) and electrospray ionization (ESI), which are used for gas chromatography–mass spectrometry (GC-MS)-based and liquid chromatography–tandem mass spectrometry (LC-MS/MS)-based compound identification, respectively.

For GC-MS-based compound identification, we used the EI mass spectra extracted from the NIST Chemistry WebBook (NIST library; http://webbook.nist.gov/chemistry/; extracted on 28 November 2011) as a reference library, and the repetitive library obtained from the NIST 08 Mass Spectral Library (NIST08/2008) as the query data. The NIST Chemistry WebBook service provides chemical and physical information for chemical compounds generated by EI mass spectrometry for users. The NIST Chemistry WebBook contained 23,721 unique compounds and their associated single mass spectra. The replicate spectral library contained 18,569 unique compounds with 28,307 mass spectra. The NIST replicate and WebBook libraries were considered as the query data and a reference library, respectively. We assumed that all of the query compounds were present in a reference library, and all of the compounds that were not present in the reference library were removed from the query data. Consequently, 12,850 unique compounds with 21,516 mass spectra remained in the query data. The m/z values were ranged from 1 to 892.

We also used the ESI mass spectra, obtained from Global Natural Products Social Molecular Networking (GNPS; https://gnps.ucsd.edu/; extracted on 2 February 2022) for LC-MS/MS-based compound identification. The GNPS database consists of 44 ESI mass spectra libraries and contains 150,798 unique compounds with 481,093 ESI mass spectra. Of these, we selected 22 GNPS libraries and restricted the search to mass spectra that were generated under positive ion mode with high quality (i.e., quality of 1), resulting in 25,576 unique compounds with 34,718 ESI mass spectra. These ESI mass spectra were further preprocessed, using the same approaches as Huber et al. [14]. That is, we limited the m/z value up to 1000 and filtered out all of the nonzero peaks with relative intensities <0.01 compared to the highest nonzero intensity peak, followed by discarding all of the mass spectra with less than 10 nonzero peaks. As a result, 14,705 unique compounds with 17,103 ESI mass spectra were left. Of these, there were 1247 compounds with 2 or more replicate ESI mass spectra and 13,458 compounds with single ESI mass spectra. The constructed library includes 13,458 compounds with single ESI mass spectra, as well as 1247 compounds with randomly chosen single ESI mass spectra, resulting in 14,705 unique compounds with their associated single mass spectra. Consequently, the query data were composed of 1247 unique compounds with 2398 ESI mass spectra. The resulted m/z values were ranged from 100 to 1000.

**Table 4:** List of all 15 binary asymmetric similarity measures.

Index	Name	Expression	Range
1	Jaccard	c/(a+b+c)	[0, 1)
2	Dice	2c/(a+b+2c)	[0, 1)
3	3W-Jaccard	3c/(a+b+3c)	[0, 1)
4	Sokal–Sneath	c/(2a+2b+c)	[0, 1)
5	Cosine	c/√((a+c)·(b+c))	[0, 1)
6	Mountford	2c/(c(a+b)+2ab)	[0, 2]
7	McConnaughey	(c^2^−ab)/((a+c)·(b+c))	[−1, 1)
8	Driver–Kroeber	c(a+b+2c)/(2(a+c)·(b+c))	[0, 1)
9	Simpson	c/min(a+c,b+c)	[0, 1)
10	Braun–Banquet	c/max(a+c,b+c)	[0, 1)
11	Fager–McGowan	c/√((a+c)·(b+c)) − 1/(2·√(max(a+c,b+c)))	(−1/2, 1)
12	Kulczynski	c/(a+b)	[0, ∞)
13	Intersection	c	[0, ∞)
14	Hamming	1/(a+b)	(0, 1]
15	Hellinger	1 − √((1 − c/√((a+c)·(b+c))))	[0, 1)

1, Jaccard is also known as Tanimoto; 2, Dice is also known as Hodgkin index, Sorenson, Czekanowski, Nei–Li, and F1-score; 5, Cosine is equal to the square root of Sorgenfrei, and is also known as Carbo index, Ochiai, Otsuka, and Fowlkes–Mallows index; 8, Driver–Kroeber is equal to 0.5 times Johnson, and is also known as Kulczynski; 14, Hamming is also known as squared-Euclidean, Canberra, Manhattan, Cityblock, and Minkowski; a,b,c ≥ 0; a+b+c > 0.

### 4.3. Compound Identification by a Mass Spectra Library

The compound identification using a mass spectra library is to identify compounds by comparing a query mass spectrum of an unknown compound against all of the reference mass spectra of a mass spectra library. Then, all of the reference compounds are ranked, based on the similarity scores with the query mass spectrum and the best matching reference compound is matched to the query mass spectrum.

To compute the similarity scores between a query mass spectrum and all of the reference mass spectra, the m/z values between two mass spectra should be matched to each other, which is called peak matching. For the EI mass spectra (i.e., GC-MS-based compound identification), no peak matching is required because the m/z values are integers and m/z does not shift. On the other hand, the m/z values of the ESI mass spectra (i.e., LC-MS/MS-based compound identification) are real numbers and shifted by differences in precursor m/z values. Thus, peak matching is required, and the m/z variation window used to match the peaks was ±0.2.

The performance of each binary similarity measure was evaluated, based on the accuracy of the compound identification. The identification accuracy was defined as the proportion of query mass spectra matched correctly as:

(1) $$\mathrm{Accuracy}=\frac{\mathrm{Number}\;\mathrm{of}\;\mathrm{query}\;\mathrm{mass}\;\mathrm{spectra}\;\mathrm{matched}\;\mathrm{correctly}}{\mathrm{Total}\;\mathrm{number}\;\mathrm{of}\;\mathrm{query}\;\mathrm{mass}\;\mathrm{spectra}}.$$

The chemical abstract service (CAS) registry number was used to identify the correct match for the NIST EI mass spectra, while the molecular name was used for the GNPS ESI mass spectra.

## 5. Conclusions

We found that the Jaccard, Dice, 3W-Jaccard, Sokal–Sneath, and Kulczynski measures have the same identification accuracy; the Cosine and Hellinger measures have the same identification accuracy; and the McConnaughey and Driver–Kroeber measures have the same identification accuracy. This observation was first confirmed theoretically and further practically confirmed, using the EI and ESI mass spectra libraries. Among the 15 selected binary asymmetric similarity measures, the best performing similarity measures were McConnaughey and Driver–Kroeber measures for the EI mass spectra and Cosine and Hellinger measures for the ESI mass spectra in compound identification, affirming that the identification performance is data-dependent. Besides, the Fager–McGowan measure was the second-best performing similarity measure in both the EI and ESI mass spectra, implying that it is data-independent and the most robust similarity measure.

Based on the results, it is recommended to use Driver–Kroeber or McConnaughey measurements for GC-MS-based untargeted metabolomics, and Cosine and Hellinger measurements for LC-MS-based untargeted metabolomics.

## Figures and Tables

**Table 1:** Accuracy and 95% CI of the 15 binary similarity measures for the EI mass spectra-based compound identification up to the top three highest similarity scores.

Similarity Measures	Ranks		
	1	2	3
1	27.49 (26.89,28.07)	35.08 (34.43,35.72)	39.41 (38.76,40.07)
2	27.49 (26.89,28.07)	35.08 (34.43,35.72)	39.41 (38.76,40.07)
3	27.49 (26.89,28.07)	35.08 (34.43,35.72)	39.41 (38.76,40.07)
4	27.49 (26.89,28.07)	35.08 (34.43,35.72)	39.41 (38.76,40.07)
5	29.11 (28.50,29.71)	37.27 (36.61,37.92)	42.03 (41.38,42.68)
6	27.51 (26.92,28.10)	35.63 (34.99,36.28)	40.19 (39.55,40.86)
7	31.24 (30.62,31.86)	40.24 (39.59,40.90)	45.36 (44.68,46.02)
8	31.24 (30.62,31.86)	40.24 (39.59,40.90)	45.36 (44.68,46.02)
9	20.71 (20.17,21.25)	20.80 (20.25,21.34)	20.90 (20.34,21.44)
10	18.32 (17.81,18.83)	23.78 (23.21,24.36)	26.65 (26.07,27.24)
11	29.78 (29.17,30.39)	38.09 (37.43,38.74)	42.88 (42.22,43.54)
12	27.49 (26.89,28.07)	35.08 (34.43,35.72)	39.41 (38.76,40.07)
13	15.21 (14.75,15.69)	15.40 (14.91,15.89)	15.60 (15.11,16.09)
14	26.16 (25.57,26.76)	33.25 (32.63,33.89)	37.34 (36.71,38.00)
15	29.11 (28.50,29.71)	37.27 (36.62,37.93)	42.03 (41.38,42.68)

The numbers in parentheses are 95% CI.

**Table 2:** Accuracy and 95% CI of all 15 binary similarity measures for ESI mass spectra-based compound identification up to top three highest similarity scores.

Similarity Measures	Ranks		
	1	2	3
1	52.24 (50.23,54.29)	59.56 (57.64,61.49)	62.83 (60.90,64.76)
2	52.24 (50.23,54.29)	59.56 (57.64,61.49)	62.83 (60.90,64.76)
3	52.24 (50.23,54.29)	59.56 (57.64,61.49)	62.83 (60.90,64.76)
4	52.24 (50.23,54.29)	59.56 (57.64,61.49)	62.83 (60.90,64.76)
5	53.37 (51.36,55.38)	60.32 (58.39,62.24)	64.13 (62.20,66.05)
6	48.01 (46.00,49.98)	54.37 (52.45,56.38)	56.72 (54.75,58.73)
7	51.15 (49.14,53.16)	58.23 (56.26,60.23)	61.87 (59.94,63.79)
8	51.15 (49.14,53.16)	58.23 (56.26,60.23)	61.87 (59.94,63.79)
9	42.78 (40.85,44.70)	45.21 (43.20,47.22)	47.59 (45.63,49.60)
10	50.31 (48.26,52.28)	57.22 (55.25,59.19)	61.07 (59.15,63.04)
11	53.33 (51.32,55.34)	60.36 (58.39,62.29)	63.83 (61.95,65.80)
12	52.24 (50.23,54.29)	59.56 (57.64,61.49)	62.83 (60.90,64.76)
13	36.12 (34.24,38.09)	39.18 (37.21,41.15)	41.23 (39.22,43.24)
14	47.34 (45.33,49.31)	52.32 (50.36,54.37)	54.46 (52.45,56.43)
15	53.37 (51.36,55.38)	60.32 (58.39,62.24)	64.13 (62.20,66.05)

The numbers in parentheses are 95% CI.

**Table 3:** A confusion matrix between binary query and reference mass spectra.

		Reference Mass Spectra	
		0	1
Query mass spectra	0	d	b
	1	a	c

‘0’ indicates that a peak intensity is zero, while ‘1’ represents a nonzero intensity.

## Data Availability

Publicly available datasets were analyzed in this study. This data can be found here: [http://webbook.nist.gov/chemistry/; https://gnps.ucsd.edu/].

## Supplementary Materials

The following supporting information can be downloaded at: https://www.mdpi.com/article/10.3390/metabo12080694/s1, Proofs of the relationships (1) and (2); Tables S1–S6; Figures S1–S8.
