# Supplementary material for: Comparative Analysis of Binary Similarity Measures for Compound Identification in Mass Spectrometry-Based Metabolomics
Source: Metabolites. 2022 Jul 26;12(8):694. doi: 10.3390/metabo12080694 (PMC9394311; doi:10.3390/metabo12080694)
Supplement: Supplementary file 1 [file metabolites-12-00694-s001.zip › metabolites-1788139-supplementary.pdf]

Binary Similarity Measures on Compound Identification

Seongho Kim [1,*], Ikuko Kato [2] and Xiang Zhang [3]

[1] Biostatistics and Bioinformatics Core, Karmanos Cancer Institute, Department of Oncology, School of Medicine, Wayne State University, Detroit, MI 48201, USA; kimse@karmanos.org
[2] Department of Oncology and Pathology, School of Medicine, Wayne State University, Detroit, MI 48201; katoi@karmanos.org
[3] Department of Chemistry, University of Louisville, Louisville, KY 40292; xiang.zhang@louisville.edu
* Correspondence: kimse@karmanos.org; Tel.: +1-313-576-8653

Supplementary Information

Contents

Proofs of the relationships (1) and (2) --- Page 1
Table S1 (using EI mass spectral library) --- Page 3
Table S2 (using EI mass spectral library) --- Page 4
Table S3 (using EI mass spectral library) --- Page 5
Table S4 (using ESI mass spectral library) --- Page 6
Table S5 (using ESI mass spectral library) --- Page 7
Table S6 (using ESI mass spectral library) --- Page 8
Figure S1 (using EI mass spectral library) --- Page 9
Figure S2 (using ESI mass spectral library) --- Page 10
Figure S3 (using EI mass spectral library) --- Page 11
Figure S4 (using EI mass spectral library) --- Page 12
Figure S5 (using ESI mass spectral library) --- Page 13
Figure S6 (using ESI mass spectral library) --- Page 14
Figure S7 (using ESI mass spectral library) --- Page 15
Figure S8 (using ESI mass spectral library) --- Page 16

Proofs of the relationships (1) and (2)

(1) The similarity measures 1 (Jaccard), 2 (Dice), 3 (3W-Jaccard), 4 (Sokal-Sneath), and 12 (Kulczynski) are strictly order-preserving.

Proof: Since $a, b, c \geq 0$ and $a + b + c > 0$, all denominators of all these five measures are greater than zero and we can derive, for each measure, the followings:

$$Measure\ 1\ (Jaccard): \frac{c_2}{a_2 + b_2 + c_2} - \frac{c_1}{a_1 + b_1 + c_1} > 0 \Leftrightarrow c_2(a_1 + b_1 + c_1) - c_1(a_2 + b_2 + c_2) > 0; \quad (A1)$$

$$Measure\ 2\ (Dice): \frac{2c_2}{a_2 + b_2 + 2c_2} - \frac{2c_1}{a_1 + b_1 + 2c_1} > 0 \Leftrightarrow 2c_2(a_1 + b_1 + 2c_1) - 2c_1(a_2 + b_2 + 2c_2) > 0; \quad (A2)$$

$$Measure\ 3\ (3W - Jaccard): \frac{3c_2}{a_2 + b_2 + 3c_2} - \frac{3c_1}{a_1 + b_1 + 3c_1} > 0 \Leftrightarrow 3c_2(a_1 + b_1 + 3c_1) - 3c_1(a_2 + b_2 + 3c_2) > 0; \quad (A3)$$

$$Measure\ 4\ (Sokal - Sneath): \frac{c_2}{2a_2 + 2b_2 + c_2} - \frac{c_1}{2a_1 + 2b_1 + c_1} > 0 \Leftrightarrow c_2(2a_1 + 2b_1 + c_1) - c_1(2a_2 + 2b_2 + c_2) > 0; \quad (A4)$$

$$Measure\ 12\ (Kulczynski): \frac{c_2}{a_2 + b_2} - \frac{c_1}{a_1 + b_1} > 0 \Leftrightarrow c_2(a_1 + b_1) - c_1(a_2 + b_2) > 0; \quad (A5)$$

Thus, it is sufficient to show the equivalence among the right-hand side inequalities. We will prove in the following orders: $(A2) \Leftrightarrow (A1), (A3) \Leftrightarrow (A1), (A4) \Leftrightarrow (A1)$, and $(A5) \Leftrightarrow (A1)$.

- $(A2) \Leftrightarrow (A1)$

$$\begin{aligned} 2c_2(a_1+b_1+2c_1) - 2c_1(a_2+b_2+2c_2) > 0 &\Leftrightarrow 2c_2(a_1+b_1+c_1) - 2c_1(a_2+b_2+c_2) + 2c_1c_2 - 2c_1c_2 > 0 \\ &\Leftrightarrow 2\{c_2(a_1+b_1+c_1) - c_1(a_2+b_2+c_2)\} > 0 \\ &\Leftrightarrow \{c_2(a_1+b_1+c_1) - c_1(a_2+b_2+c_2)\} > 0 \end{aligned}$$

- $(A3) \Leftrightarrow (A1)$

$$\begin{aligned} 3c_2(a_1+b_1+3c_1) - 3c_1(a_2+b_2+3c_2) > 0 &\Leftrightarrow 3c_2(a_1+b_1+c_1) - 3c_1(a_2+b_2+c_2) + 6c_1c_2 - 6c_1c_2 > 0 \\ &\Leftrightarrow 3\{c_2(a_1+b_1+c_1) - c_1(a_2+b_2+c_2)\} > 0 \\ &\Leftrightarrow \{c_2(a_1+b_1+c_1) - c_1(a_2+b_2+c_2)\} > 0 \end{aligned}$$

- $(A4) \Leftrightarrow (A1)$

$$\begin{aligned} c_2(2a_1+2b_1+c_1) - c_1(2a_2+2b_2+c_2) > 0 &\Leftrightarrow c_2(2a_1+2b_1+2c_1-c_1) - c_1(2a_2+2b_2+2c_2-c_2) > 0 \\ &\Leftrightarrow 2\{c_2(a_1+b_1+c_1) - c_1(a_2+b_2+c_2)\} - c_2c_1 + c_1c_2 > 0 \\ &\Leftrightarrow 2\{c_2(a_1+b_1+c_1) - c_1(a_2+b_2+c_2)\} > 0 \\ &\Leftrightarrow \{c_2(a_1+b_1+c_1) - c_1(a_2+b_2+c_2)\} > 0 \end{aligned}$$

- $(A5) \Leftrightarrow (A1)$

$$\begin{aligned} c_2(a_1+b_1) - c_1(a_2+b_2) > 0 &\Leftrightarrow c_2(a_1+b_1+c_1-c_1) - c_1(a_2+b_2+c_2-c_2) > 0 \\ &\Leftrightarrow \{c_2(a_1+b_1+c_1) - c_1(a_2+b_2+c_2)\} - c_2c_1 + c_1c_2 > 0 \\ &\Leftrightarrow \{c_2(a_1+b_1+c_1) - c_1(a_2+b_2+c_2)\} > 0 \end{aligned}$$

Thus, $(A1) \Leftrightarrow (A2) \Leftrightarrow (A3) \Leftrightarrow (A4) \Leftrightarrow (A5)$ and the proof is completed. □

(2) The similarity measures 5 (Cosine) and 15 (Hellinger) are strictly order-preserving.

Proof: Since $a, b, c \geq 0$ and $a+b+c > 0$, the denominator, $\sqrt{(a+c)(b+c)}$, of these two measures are greater than zero and we can derive the followings:

$$Measure\ 15\ (Hellinger): 1 - \sqrt{1 - \frac{c_2}{\sqrt{(a_2+c_2)(b_2+c_2)}}} - 1 + \sqrt{1 - \frac{c_1}{\sqrt{(a_1+c_1)(b_1+c_1)}}} > 0 \quad (A6)$$

$$\Leftrightarrow \sqrt{1 - \frac{c_1}{\sqrt{(a_1+c_1)(b_1+c_1)}}} > \sqrt{1 - \frac{c_2}{\sqrt{(a_2+c_2)(b_2+c_2)}}} \quad (A7)$$

$$\Leftrightarrow 1 - \frac{c_1}{\sqrt{(a_1+c_1)(b_1+c_1)}} > 1 - \frac{c_2}{\sqrt{(a_2+c_2)(b_2+c_2)}} \quad (A8)$$

$$\Leftrightarrow \frac{c_2}{\sqrt{(a_2+c_2)(b_2+c_2)}} - \frac{c_1}{\sqrt{(a_1+c_1)(b_1+c_1)}} > 0: Measure\ 5\ (Cosine) \quad (A9)$$

Thus, $(A6) \Leftrightarrow (A9)$ and the proof is completed. □

Table S1. Pearson's correlation matrix of similarity scores among 15 similarity measures

Similarity measures (columns) × Similarity meausres (rows)

	14	9	13	6	8	7	11	5	15	12	10	3	2	4	1
14		0.0649	-0.1273	0.8355	0.2948	0.2948	0.3232	0.3820	0.4145	0.5237	0.4825	0.4050	0.4307	0.4905	0.4662
9	0.0649		0.5911	0.5358	0.8852	0.8852	0.7757	0.7522	0.7310	0.5266	0.4616	0.6471	0.6359	0.5891	0.6129
13	-0.1273	0.5911		0.2232	0.7352	0.7352	0.7775	0.7490	0.7553	0.6868	0.6769	0.7233	0.7322	0.7295	0.7359
6	0.8355	0.5358	0.2232		0.6987	0.6987	0.6851	0.7164	0.7415	0.7669	0.6752	0.6848	0.7065	0.7516	0.7345
8	0.2948	0.8852	0.7352	0.6987		1.0000	0.9759	0.9692	0.9594	0.8079	0.8213	0.9184	0.9170	0.8824	0.9037
7	0.2948	0.8852	0.7352	0.6987	1.0000		0.9759	0.9692	0.9594	0.8079	0.8213	0.9184	0.9170	0.8824	0.9037
11	0.3232	0.7757	0.7775	0.6851	0.9759	0.9759		0.9975	0.9913	0.8628	0.9095	0.9775	0.9779	0.9431	0.9653
5	0.3820	0.7522	0.7490	0.7164	0.9692	0.9692	0.9975		0.9947	0.8722	0.9257	0.9856	0.9866	0.9527	0.9747
15	0.4145	0.7310	0.7553	0.7415	0.9594	0.9594	0.9913	0.9947		0.9134	0.9330	0.9761	0.9854	0.9756	0.9868
12	0.5237	0.5266	0.6868	0.7669	0.8079	0.8079	0.8628	0.8722	0.9134		0.8946	0.8555	0.8904	0.9673	0.9370
10	0.4825	0.4616	0.6769	0.6752	0.8213	0.8213	0.9095	0.9257	0.9330	0.8946		0.9576	0.9688	0.9601	0.9715
3	0.4050	0.6471	0.7233	0.6848	0.9184	0.9184	0.9775	0.9856	0.9761	0.8555	0.9576		0.9963	0.9476	0.9763
2	0.4307	0.6359	0.7322	0.7065	0.9170	0.9170	0.9779	0.9866	0.9854	0.8904	0.9688	0.9963		0.9706	0.9912
4	0.4905	0.5891	0.7295	0.7516	0.8824	0.8824	0.9431	0.9527	0.9756	0.9673	0.9601	0.9476	0.9706		0.9937
1	0.4662	0.6129	0.7359	0.7345	0.9037	0.9037	0.9653	0.9747	0.9868	0.9370	0.9715	0.9763	0.9912	0.9937	

Table S2. Identification accuracy (%) and their associated 95% confidence intervals for all similarity measures by rank

Similarity meausres	Rank 1	2	3	4	5	10
1	27.49 (26.89,28.07)	35.08 (34.43,35.72)	39.41 (38.76,40.07)	42.03 (41.38,42.69)	43.93 (43.26,44.60)	50.13 (49.48,50.79)
2	27.49 (26.89,28.07)	35.08 (34.43,35.72)	39.41 (38.76,40.07)	42.03 (41.38,42.69)	43.93 (43.26,44.60)	50.13 (49.48,50.79)
3	27.49 (26.89,28.07)	35.08 (34.43,35.72)	39.41 (38.76,40.07)	42.03 (41.38,42.69)	43.93 (43.26,44.60)	50.13 (49.48,50.79)
4	27.49 (26.89,28.07)	35.08 (34.43,35.72)	39.41 (38.76,40.07)	42.03 (41.38,42.69)	43.93 (43.26,44.60)	50.13 (49.48,50.79)
5	29.11 (28.50,29.71)	37.27 (36.61,37.92)	42.03 (41.38,42.68)	44.96 (44.31,45.63)	47.10 (46.44,47.79)	53.64 (52.99,54.31)
6	27.51 (26.92,28.10)	35.63 (34.99,36.28)	40.19 (39.55,40.86)	43.18 (42.55,43.86)	45.47 (44.80,46.13)	52.27 (51.64,52.95)
7	31.24 (30.62,31.86)	40.24 (39.59,40.90)	45.36 (44.68,46.02)	48.39 (47.73,49.07)	50.53 (49.86,51.19)	57.32 (56.67,57.99)
8	31.24 (30.62,31.86)	40.24 (39.59,40.90)	45.36 (44.68,46.02)	48.39 (47.73,49.07)	50.53 (49.86,51.19)	57.32 (56.67,57.99)
9	20.71 (20.17,21.25)	20.80 (20.25,21.34)	20.90 (20.34,21.44)	20.95 (20.41,21.49)	21.02 (20.49,21.55)	21.43 (20.88,21.97)
10	18.32 (17.81,18.83)	23.78 (23.21,24.36)	26.65 (26.07,27.24)	28.83 (28.24,29.42)	30.32 (29.70,30.95)	34.92 (34.29,35.56)
11	29.78 (29.17,30.39)	38.09 (37.43,38.74)	42.88 (42.22,43.54)	45.96 (45.31,46.63)	48.02 (47.36,48.69)	54.72 (54.06,55.38)
12	27.49 (26.89,28.07)	35.08 (34.43,35.72)	39.41 (38.76,40.07)	42.03 (41.38,42.69)	43.93 (43.26,44.60)	50.13 (49.48,50.79)
13	15.21 (14.75,15.69)	15.40 (14.91,15.89)	15.60 (15.11,16.09)	15.76 (15.29,16.25)	15.96 (15.47,16.44)	16.65 (16.16,17.15)
14	26.16 (25.57,26.76)	33.25 (32.63,33.89)	37.34 (36.71,38.00)	39.92 (39.28,40.57)	41.65 (40.99,42.33)	46.74 (46.10,47.43)
15	29.11 (28.50,29.71)	37.27 (36.62,37.93)	42.03 (41.38,42.68)	44.96 (44.31,45.63)	47.10 (46.44,47.79)	53.64 (52.99,54.31)

Similarity meausres	Rank 15	20	30	50	100	500
1	53.51 (52.83,54.19)	56.06 (55.39,56.73)	59.51 (58.85,60.16)	63.67 (63.02,64.30)	69.57 (68.97,70.19)	81.78 (81.27,82.29)
2	53.51 (52.83,54.19)	56.06 (55.39,56.73)	59.51 (58.85,60.16)	63.67 (63.02,64.30)	69.57 (68.97,70.19)	81.78 (81.27,82.29)
3	53.51 (52.83,54.19)	56.06 (55.39,56.73)	59.51 (58.85,60.16)	63.67 (63.02,64.30)	69.57 (68.97,70.19)	81.78 (81.27,82.29)
4	53.51 (52.83,54.19)	56.06 (55.39,56.73)	59.51 (58.85,60.16)	63.67 (63.02,64.30)	69.57 (68.97,70.19)	81.78 (81.27,82.29)
5	57.32 (56.64,57.99)	60.04 (59.38,60.69)	63.61 (62.97,64.25)	67.93 (67.29,68.54)	73.53 (72.94,74.11)	85.36 (84.89,85.83)
6	56.01 (55.33,56.67)	58.71 (58.07,59.37)	62.54 (61.90,63.19)	67.36 (66.74,67.98)	73.93 (73.36,74.51)	87.35 (86.91,87.79)
7	60.98 (60.32,61.65)	63.76 (63.13,64.40)	67.29 (66.67,67.91)	71.54 (70.93,72.13)	76.95 (76.39,77.51)	87.79 (87.35,88.22)
8	60.98 (60.32,61.65)	63.76 (63.13,64.40)	67.29 (66.67,67.91)	71.54 (70.93,72.13)	76.95 (76.39,77.51)	87.79 (87.35,88.22)
9	21.90 (21.35,22.45)	22.37 (21.80,22.93)	23.31 (22.75,23.87)	25.30 (24.72,25.87)	30.23 (29.63,30.85)	54.96 (54.31,55.63)
10	37.65 (36.99,38.31)	39.88 (39.21,40.54)	42.91 (42.25,43.56)	46.82 (46.15,47.49)	52.52 (51.86,53.19)	67.45 (66.84,68.08)
11	58.44 (57.76,59.11)	61.01 (60.36,61.65)	64.51 (63.88,65.16)	68.73 (68.10,69.34)	74.27 (73.69,74.86)	85.61 (85.13,86.07)
12	53.51 (52.83,54.19)	56.06 (55.39,56.73)	59.51 (58.85,60.16)	63.67 (63.02,64.30)	69.57 (68.97,70.19)	81.78 (81.27,82.29)
13	17.25 (16.75,17.75)	17.91 (17.39,18.41)	19.18 (18.66,19.71)	21.50 (20.95,22.06)	25.79 (25.21,26.38)	43.74 (43.09,44.39)
14	49.80 (49.13,50.48)	51.87 (51.20,52.53)	54.81 (54.14,55.47)	58.38 (57.71,59.03)	63.13 (62.50,63.77)	74.44 (73.86,75.01)
15	57.32 (56.64,57.99)	60.04 (59.38,60.69)	63.61 (62.97,64.25)	67.93 (67.29,68.54)	73.53 (72.94,74.11)	85.36 (84.89,85.83)

Table S3. Pearson's correlation matrix of identification results of rank 1 among 15 similarity measures

Similarity meausres	Similarity measures: 9	13	10	14	6	7	8	11	5	15	12	4	3	1	2
**9**	1.0000	0.8151	-0.1495	-0.1429	-0.1122	-0.0582	-0.0582	-0.0906	-0.1038	-0.1038	-0.1215	-0.1215	-0.1215	-0.1215	-0.1215
**13**	0.8151	1.0000	-0.1250	-0.1388	-0.1085	-0.0177	-0.0177	-0.0503	-0.0683	-0.0683	-0.0918	-0.0918	-0.0918	-0.0918	-0.0918
**10**	-0.1495	-0.1250	1.0000	0.7070	0.6522	0.5988	0.5988	0.6262	0.6499	0.6499	0.6884	0.6884	0.6884	0.6884	0.6884
**14**	-0.1429	-0.1388	0.7070	1.0000	0.8959	0.7512	0.7512	0.7574	0.7955	0.7955	0.8252	0.8252	0.8252	0.8252	0.8252
**6**	-0.1122	-0.1085	0.6522	0.8959	1.0000	0.7983	0.7983	0.8046	0.8411	0.8411	0.8633	0.8633	0.8633	0.8633	0.8633
**7**	-0.0582	-0.0177	0.5988	0.7512	0.7983	1.0000	1.0000	0.9355	0.9179	0.9179	0.8693	0.8693	0.8693	0.8693	0.8693
**8**	-0.0582	-0.0177	0.5988	0.7512	0.7983	1.0000	1.0000	0.9355	0.9179	0.9179	0.8693	0.8693	0.8693	0.8693	0.8693
**11**	-0.0906	-0.0503	0.6262	0.7574	0.8046	0.9355	0.9355	1.0000	0.9580	0.9580	0.9087	0.9087	0.9087	0.9087	0.9087
**5**	-0.1038	-0.0683	0.6499	0.7955	0.8411	0.9179	0.9179	0.9580	1.0000	1.0000	0.9448	0.9448	0.9448	0.9448	0.9448
**15**	-0.1038	-0.0683	0.6499	0.7955	0.8411	0.9179	0.9179	0.9580	1.0000	1.0000	0.9448	0.9448	0.9448	0.9448	0.9448
**12**	-0.1215	-0.0918	0.6884	0.8252	0.8633	0.8693	0.8693	0.9087	0.9448	0.9448	1.0000	1.0000	1.0000	1.0000	1.0000
**4**	-0.1215	-0.0918	0.6884	0.8252	0.8633	0.8693	0.8693	0.9087	0.9448	0.9448	1.0000	1.0000	1.0000	1.0000	1.0000
**3**	-0.1215	-0.0918	0.6884	0.8252	0.8633	0.8693	0.8693	0.9087	0.9448	0.9448	1.0000	1.0000	1.0000	1.0000	1.0000
**1**	-0.1215	-0.0918	0.6884	0.8252	0.8633	0.8693	0.8693	0.9087	0.9448	0.9448	1.0000	1.0000	1.0000	1.0000	1.0000
**2**	-0.1215	-0.0918	0.6884	0.8252	0.8633	0.8693	0.8693	0.9087	0.9448	0.9448	1.0000	1.0000	1.0000	1.0000	1.0000

Table S4. Pearson's correlation matrix of similarity scores among 15 similarity measures

Similarity measures	Similarity measures: 14	6	9	8	7	13	12	11	5	15	3	2	10	4	1
14		0.3124	-0.2389	-0.2117	-0.2117	-0.2304	0.0607	-0.4660	-0.1250	-0.1074	-0.0786	-0.0625	-0.0297	-0.0144	-0.0374
6	0.3124		0.3325	0.3909	0.3909	0.0890	0.5957	0.3498	0.4567	0.4795	0.4392	0.4556	0.4465	0.5181	0.4855
9	-0.2389	0.3325		0.9790	0.9790	0.5100	0.3966	0.8041	0.7906	0.7728	0.6291	0.6158	0.5152	0.5569	0.5891
8	-0.2117	0.3909	0.9790		1.0000	0.5891	0.5142	0.8805	0.8942	0.8809	0.7701	0.7607	0.6792	0.7055	0.7378
7	-0.2117	0.3909	0.9790	1.0000		0.5891	0.5142	0.8805	0.8942	0.8809	0.7701	0.7607	0.6792	0.7055	0.7378
13	-0.2304	0.0890	0.5100	0.5891	0.5891		0.4958	0.6865	0.6773	0.6824	0.6596	0.6676	0.6403	0.6538	0.6692
12	0.0607	0.5957	0.3966	0.5142	0.5142	0.4958		0.5977	0.6775	0.7170	0.6899	0.7222	0.7349	0.8257	0.7755
11	-0.4660	0.3498	0.8041	0.8805	0.8805	0.6865	0.5977		0.9281	0.9194	0.8752	0.8683	0.8065	0.8132	0.8470
5	-0.1250	0.4567	0.7906	0.8942	0.8942	0.6773	0.6775	0.9281		0.9959	0.9690	0.9661	0.9127	0.9150	0.9484
15	-0.1074	0.4795	0.7728	0.8809	0.8809	0.6824	0.7170	0.9194	0.9959		0.9654	0.9691	0.9206	0.9416	0.9632
3	-0.0786	0.4392	0.6291	0.7701	0.7701	0.6596	0.6899	0.8752	0.9690	0.9654		0.9966	0.9722	0.9394	0.9765
2	-0.0625	0.4556	0.6158	0.7607	0.7607	0.6676	0.7222	0.8683	0.9661	0.9691	0.9966		0.9807	0.9625	0.9906
10	-0.0297	0.4465	0.5152	0.6792	0.6792	0.6403	0.7349	0.8065	0.9127	0.9206	0.9722	0.9807		0.9606	0.9802
4	-0.0144	0.5181	0.5569	0.7055	0.7055	0.6538	0.8257	0.8132	0.9150	0.9416	0.9394	0.9625	0.9606		0.9898
1	-0.0374	0.4855	0.5891	0.7378	0.7378	0.6692	0.7755	0.8470	0.9484	0.9632	0.9765	0.9906	0.9802	0.9898	

Row label: Similarity meausres

Table S5. Identification accuracy (%) and their associated 95% confidence intervals for all similarity measures by rank

Similarity measures	Rank 1	Rank 2	Rank 3	Rank 4	Rank 5	Rank 10
**1**	52.24 (50.23,54.29)	59.56 (57.64,61.49)	62.83 (60.90,64.76)	65.42 (63.50,67.35)	66.60 (64.71,68.44)	71.62 (69.78,73.38)
**2**	52.24 (50.23,54.29)	59.56 (57.64,61.49)	62.83 (60.90,64.76)	65.42 (63.50,67.35)	66.60 (64.71,68.44)	71.62 (69.78,73.38)
**3**	52.24 (50.23,54.29)	59.56 (57.64,61.49)	62.83 (60.90,64.76)	65.42 (63.50,67.35)	66.60 (64.71,68.44)	71.62 (69.78,73.38)
**4**	52.24 (50.23,54.29)	59.56 (57.64,61.49)	62.83 (60.90,64.76)	65.42 (63.50,67.35)	66.60 (64.71,68.44)	71.62 (69.78,73.38)
**5**	53.37 (51.36,55.38)	60.32 (58.39,62.24)	64.13 (62.20,66.05)	66.85 (64.96,68.82)	68.35 (66.51,70.15)	73.42 (71.66,75.14)
**6**	48.01 (46.00,49.98)	54.37 (52.45,56.38)	56.72 (54.75,58.73)	59.23 (57.26,61.20)	60.74 (58.81,62.70)	65.38 (63.46,67.27)
**7**	51.15 (49.14,53.16)	58.23 (56.26,60.23)	61.87 (59.94,63.79)	64.34 (62.41,66.26)	66.14 (64.21,68.02)	70.32 (68.48,72.12)
**8**	51.15 (49.14,53.16)	58.23 (56.26,60.23)	61.87 (59.94,63.79)	64.34 (62.41,66.26)	66.14 (64.21,68.02)	70.32 (68.48,72.12)
**9**	42.78 (40.85,44.70)	45.21 (43.20,47.22)	47.59 (45.63,49.60)	49.43 (47.43,51.44)	50.94 (48.89,52.95)	55.13 (53.12,57.14)
**10**	50.31 (48.26,52.28)	57.22 (55.25,59.19)	61.07 (59.15,63.04)	63.46 (61.53,65.42)	65.09 (63.16,66.93)	69.69 (67.81,71.49)
**11**	53.33 (51.32,55.34)	60.36 (58.39,62.29)	63.83 (61.95,65.80)	66.76 (64.84,68.69)	68.19 (66.30,70.03)	73.96 (72.21,75.68)
**12**	52.24 (50.23,54.29)	59.56 (57.64,61.49)	62.83 (60.90,64.76)	65.42 (63.50,67.35)	66.60 (64.71,68.44)	71.62 (69.78,73.38)
**13**	36.12 (34.24,38.09)	39.18 (37.21,41.15)	41.23 (39.22,43.24)	43.78 (41.77,45.79)	45.33 (43.37,47.34)	49.14 (47.09,51.07)
**14**	47.34 (45.33,49.31)	52.32 (50.36,54.37)	54.46 (52.45,56.43)	55.50 (53.54,57.47)	56.30 (54.33,58.27)	58.02 (56.05,59.98)
**15**	53.37 (51.36,55.38)	60.32 (58.39,62.24)	64.13 (62.20,66.05)	66.85 (64.96,68.82)	68.35 (66.51,70.15)	73.42 (71.66,75.14)

Similarity measures	Rank 15	Rank 20	Rank 30	Rank 50	Rank 100	Rank 500
**1**	73.88 (72.16,75.64)	75.09 (73.34,76.81)	76.48 (74.76,78.15)	77.94 (76.27,79.57)	80.37 (78.74,81.96)	84.81 (83.34,86.23)
**2**	73.88 (72.16,75.64)	75.09 (73.34,76.81)	76.48 (74.76,78.15)	77.94 (76.27,79.57)	80.37 (78.74,81.96)	84.81 (83.34,86.23)
**3**	73.88 (72.16,75.64)	75.09 (73.34,76.81)	76.48 (74.76,78.15)	77.94 (76.27,79.57)	80.37 (78.74,81.96)	84.81 (83.34,86.23)
**4**	73.88 (72.16,75.64)	75.09 (73.34,76.81)	76.48 (74.76,78.15)	77.94 (76.27,79.57)	80.37 (78.74,81.96)	84.81 (83.34,86.23)
**5**	76.02 (74.34,77.73)	77.02 (75.30,78.65)	78.48 (76.85,80.12)	80.03 (78.40,81.62)	81.96 (80.37,83.47)	87.19 (85.81,88.53)
**6**	67.27 (65.42,69.19)	68.31 (66.43,70.15)	70.24 (68.35,72.04)	73.04 (71.24,74.84)	76.27 (74.59,77.94)	83.51 (82.00,84.97)
**7**	72.42 (70.62,74.22)	73.67 (71.91,75.43)	75.01 (73.25,76.77)	77.10 (75.43,78.78)	79.11 (77.48,80.75)	85.52 (84.05,86.94)
**8**	72.42 (70.62,74.22)	73.67 (71.91,75.43)	75.01 (73.25,76.77)	77.10 (75.43,78.78)	79.11 (77.48,80.75)	85.52 (84.05,86.94)
**9**	58.06 (56.05,60.07)	59.56 (57.56,61.57)	62.37 (60.40,64.29)	64.96 (63.00,66.85)	69.74 (67.89,71.62)	80.91 (79.32,82.50)
**10**	71.95 (70.15,73.75)	72.79 (70.99,74.55)	74.30 (72.50,76.06)	75.72 (74.01,77.40)	78.23 (76.56,79.91)	84.43 (82.92,85.89)
**11**	76.31 (74.63,78.02)	77.56 (75.89,79.24)	78.99 (77.40,80.58)	80.45 (78.86,82.00)	81.92 (80.37,83.47)	86.02 (84.60,87.40)
**12**	73.88 (72.16,75.64)	75.09 (73.34,76.81)	76.48 (74.76,78.15)	77.94 (76.27,79.57)	80.37 (78.74,81.96)	84.81 (83.34,86.23)
**13**	50.82 (48.81,52.83)	51.78 (49.77,53.79)	53.75 (51.78,55.76)	56.63 (54.63,58.60)	61.11 (59.23,63.12)	74.26 (72.50,76.02)
**14**	58.52 (56.55,60.57)	58.94 (56.93,60.90)	59.69 (57.72,61.66)	60.23 (58.31,62.16)	61.36 (59.44,63.33)	64.00 (62.08,65.93)
**15**	76.02 (74.34,77.73)	77.02 (75.30,78.65)	78.48 (76.85,80.12)	80.03 (78.40,81.62)	81.96 (80.37,83.47)	87.19 (85.81,88.53)

Table S6. Pearson's correlation matrix of identification results of rank 1 among 15 similarity measures

Similarity meausres	Similarity measures														
	**13**	**9**	**10**	**6**	**14**	**7**	**8**	**11**	**5**	**15**	**12**	**4**	**3**	**1**	**2**
**13**	1.0000	0.7560	0.4129	0.4999	0.4501	0.4972	0.4972	0.4601	0.4624	0.4624	0.4435	0.4435	0.4435	0.4435	0.4435
**9**	0.7560	1.0000	0.4068	0.6212	0.5596	0.5824	0.5824	0.5189	0.5250	0.5250	0.4874	0.4874	0.4874	0.4874	0.4874
**10**	0.4129	0.4068	1.0000	0.7369	0.7825	0.7622	0.7622	0.8101	0.8200	0.8200	0.8635	0.8635	0.8635	0.8635	0.8635
**6**	0.4999	0.6212	0.7369	1.0000	0.9029	0.8890	0.8890	0.8396	0.8539	0.8539	0.8379	0.8379	0.8379	0.8379	0.8379
**14**	0.4501	0.5596	0.7825	0.9029	1.0000	0.9027	0.9027	0.8644	0.8771	0.8771	0.8630	0.8630	0.8630	0.8630	0.8630
**7**	0.4972	0.5824	0.7622	0.8890	0.9027	1.0000	1.0000	0.9261	0.9275	0.9275	0.8854	0.8854	0.8854	0.8854	0.8854
**8**	0.4972	0.5824	0.7622	0.8890	0.9027	1.0000	1.0000	0.9261	0.9275	0.9275	0.8854	0.8854	0.8854	0.8854	0.8854
**11**	0.4601	0.5189	0.8101	0.8396	0.8644	0.9261	0.9261	1.0000	0.9832	0.9832	0.9414	0.9414	0.9414	0.9414	0.9414
**5**	0.4624	0.5250	0.8200	0.8539	0.8771	0.9275	0.9275	0.9832	1.0000	1.0000	0.9531	0.9531	0.9531	0.9531	0.9531
**15**	0.4624	0.5250	0.8200	0.8539	0.8771	0.9275	0.9275	0.9832	1.0000	1.0000	0.9531	0.9531	0.9531	0.9531	0.9531
**12**	0.4435	0.4874	0.8635	0.8379	0.8630	0.8854	0.8854	0.9414	0.9531	0.9531	1.0000	1.0000	1.0000	1.0000	1.0000
**4**	0.4435	0.4874	0.8635	0.8379	0.8630	0.8854	0.8854	0.9414	0.9531	0.9531	1.0000	1.0000	1.0000	1.0000	1.0000
**3**	0.4435	0.4874	0.8635	0.8379	0.8630	0.8854	0.8854	0.9414	0.9531	0.9531	1.0000	1.0000	1.0000	1.0000	1.0000
**1**	0.4435	0.4874	0.8635	0.8379	0.8630	0.8854	0.8854	0.9414	0.9531	0.9531	1.0000	1.0000	1.0000	1.0000	1.0000
**2**	0.4435	0.4874	0.8635	0.8379	0.8630	0.8854	0.8854	0.9414	0.9531	0.9531	1.0000	1.0000	1.0000	1.0000	1.0000

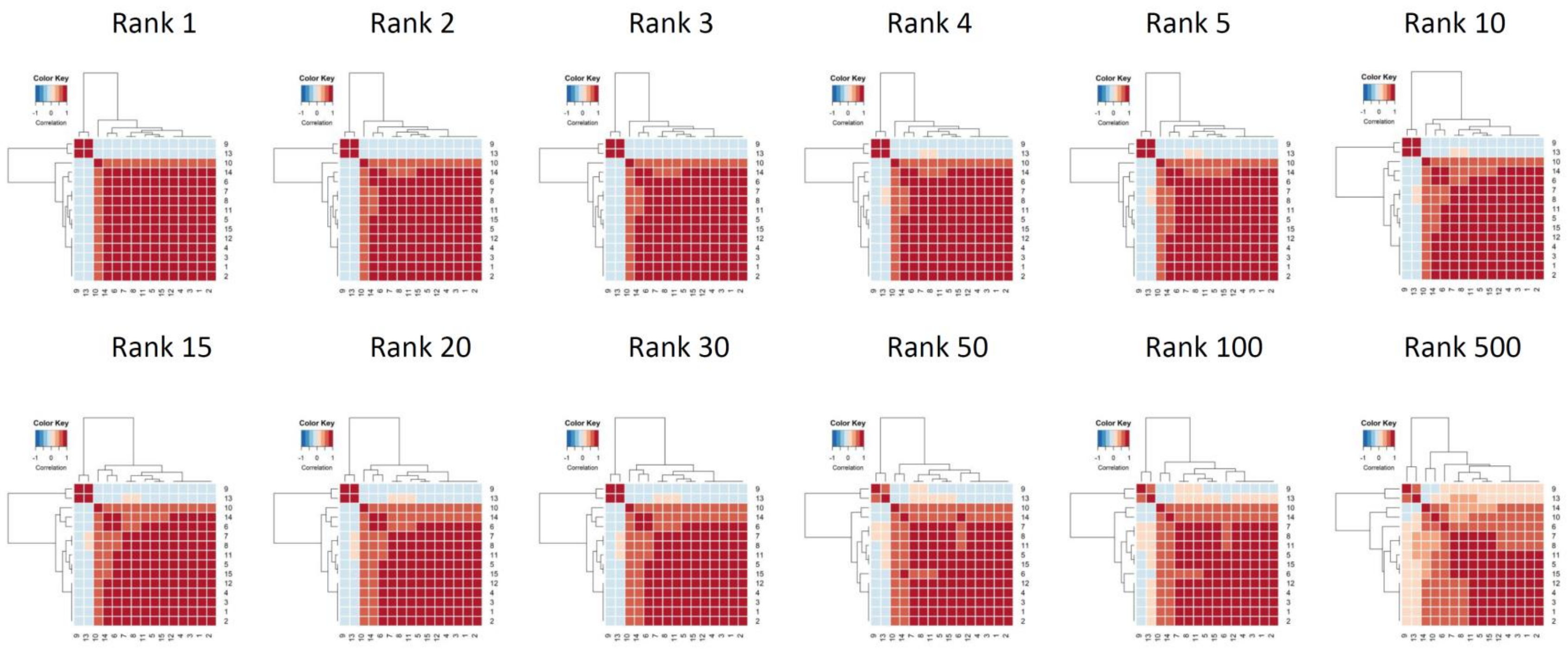

Figure S1. Heatmap of identification accuracy of all similarity scores by rank

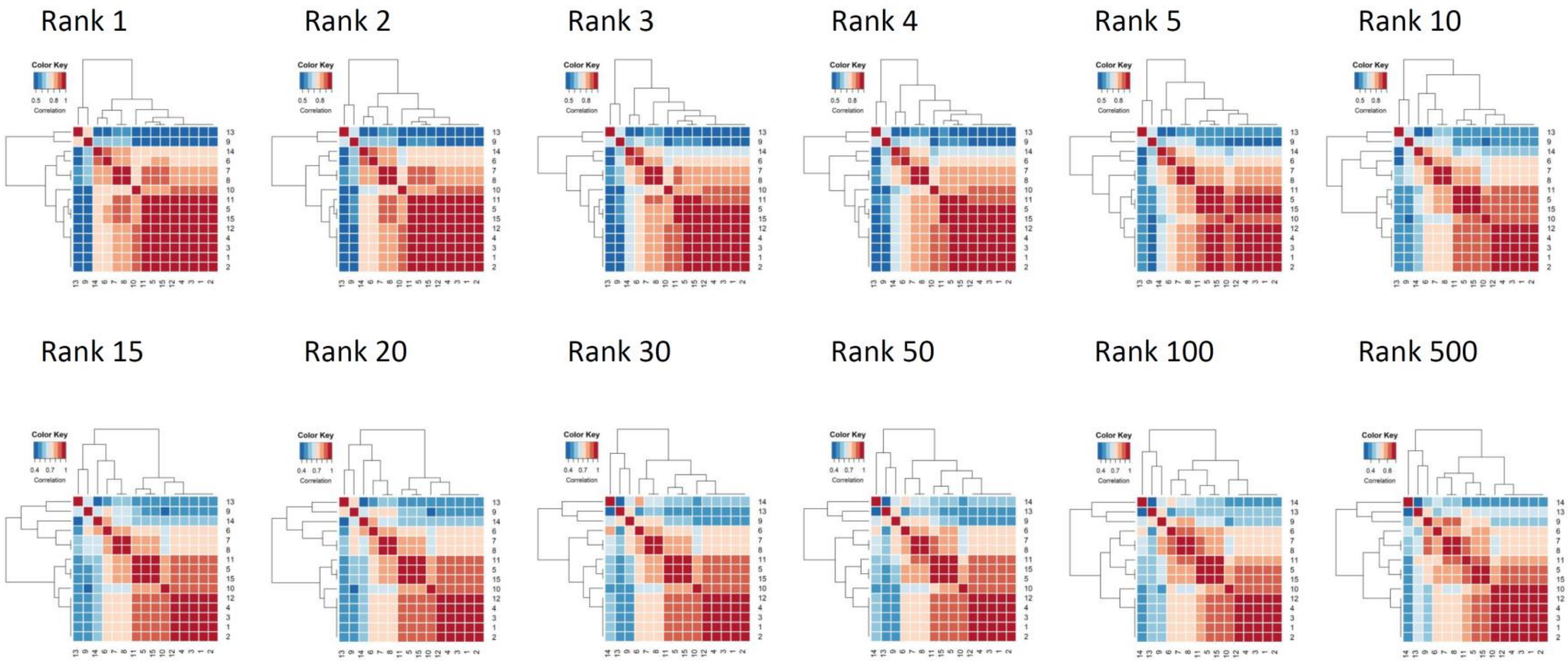

Figure S2. Heatmap of identification accuracy of all similarity scores by rank

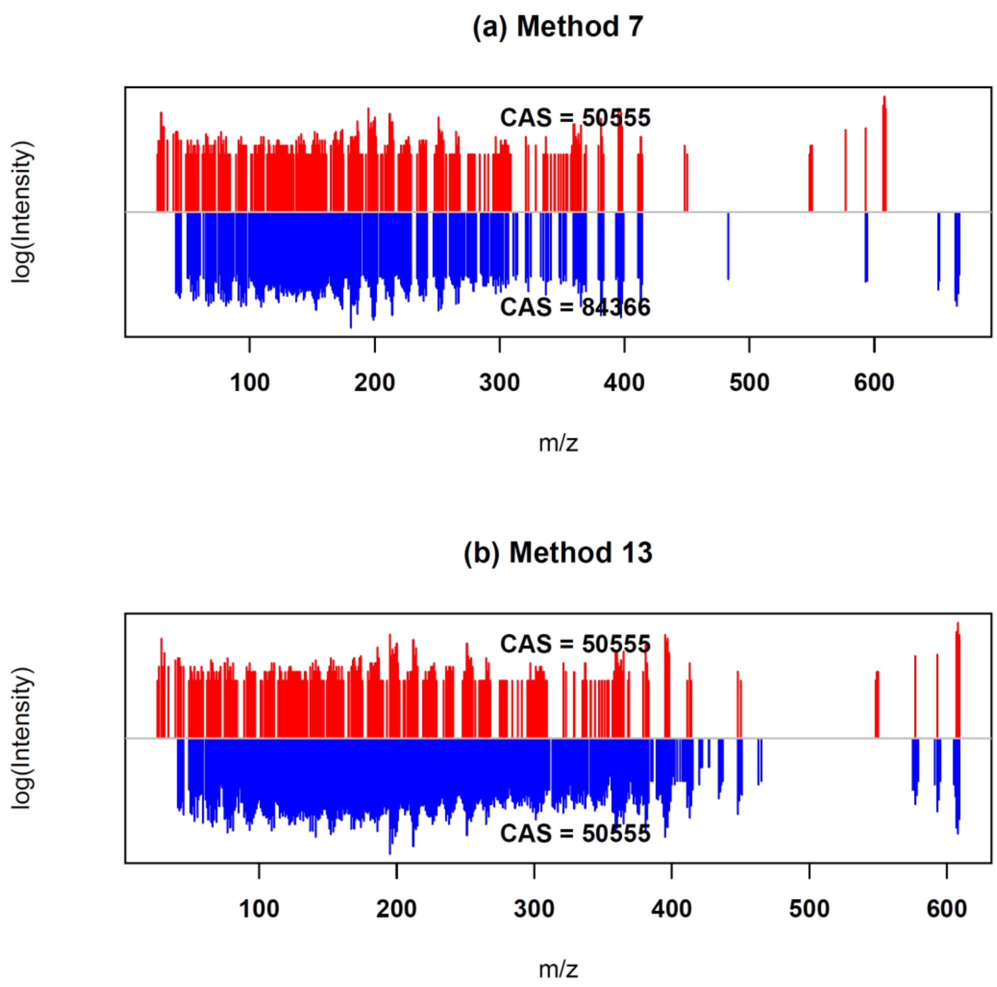

Figure S3. A case that Method 7 (Mcconnaughey) matches incorrectly while Method 13 (Intersection) matches correctly for EI. The red bars represent the query spectrum and the blue bars the library spectrum with the highest similarity score correponding to the binary similarity measure.

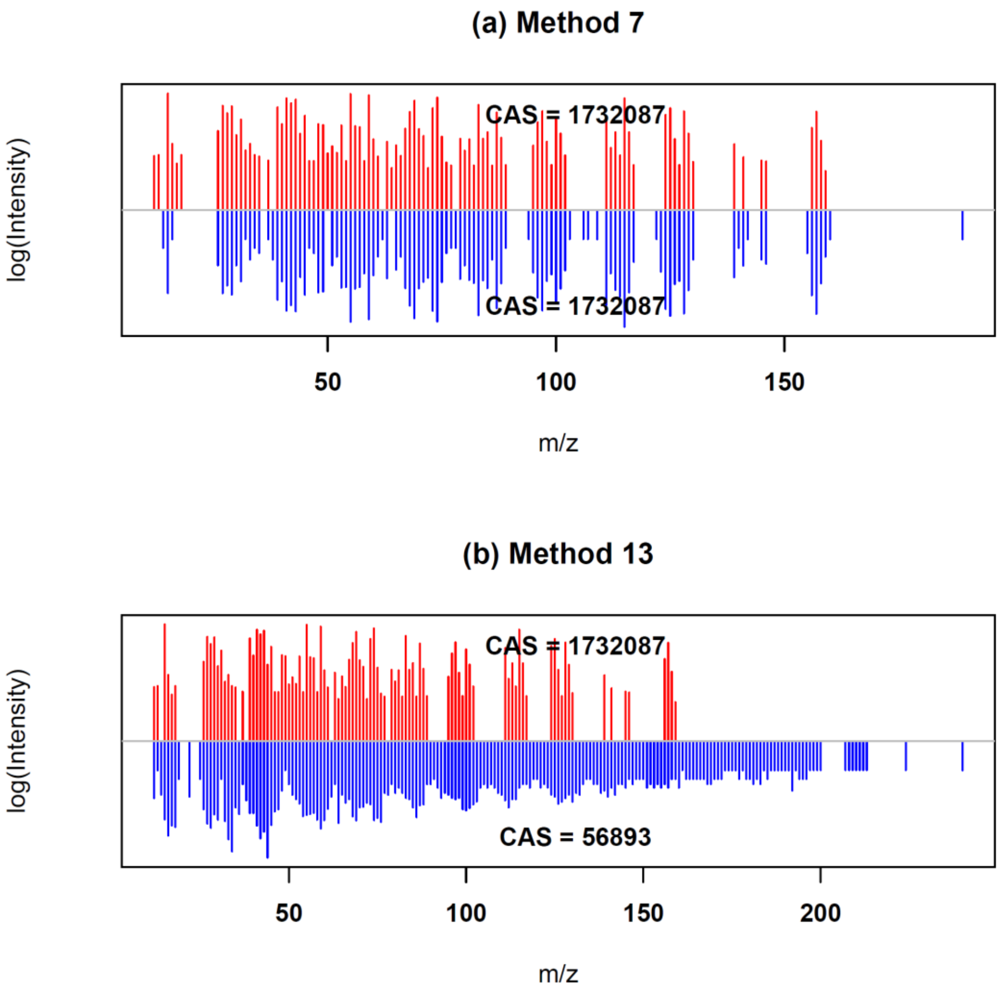

Figure S4. A case that Method 7 (Mcconnaughey) matches correctly while Method 13 (Intersection) matches incorrectly for EI. The red bars represent the query spectrum and the blue bars the library spectrum with the highest similarity score correponding to the binary similarity measure.

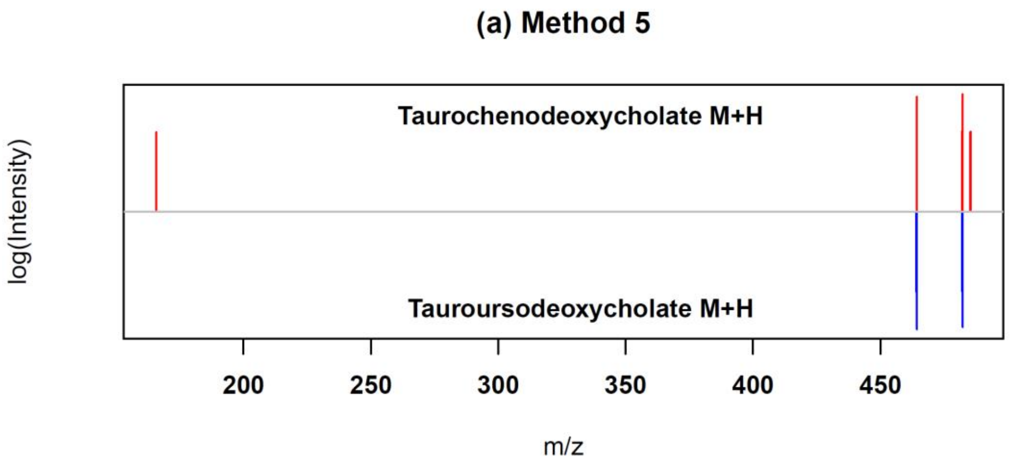

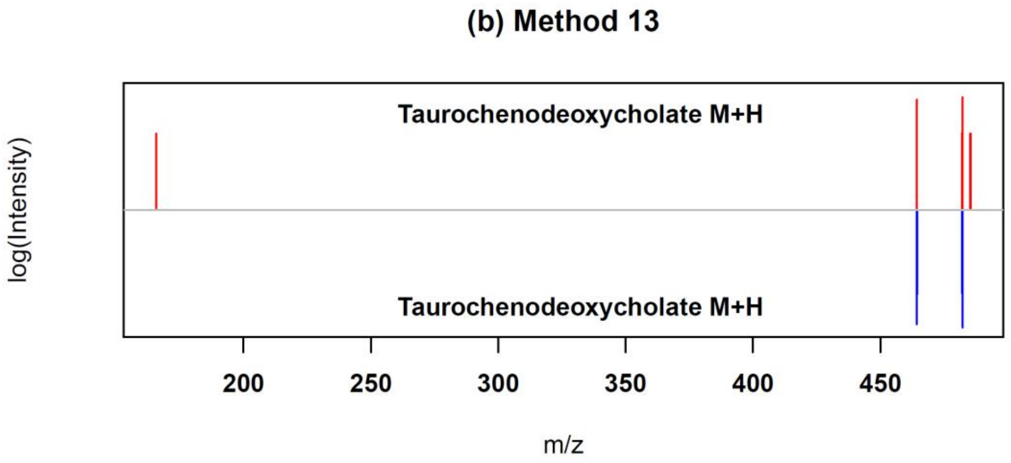

Figure S5. A case that Method 5 (Cosine) matches incorrectly while Method 13 (Intersection) matches correctly for ESI. The red bars represent the query spectrum and the blue bars the library spectrum with the highest similarity score correponding to the binary similarity measure.

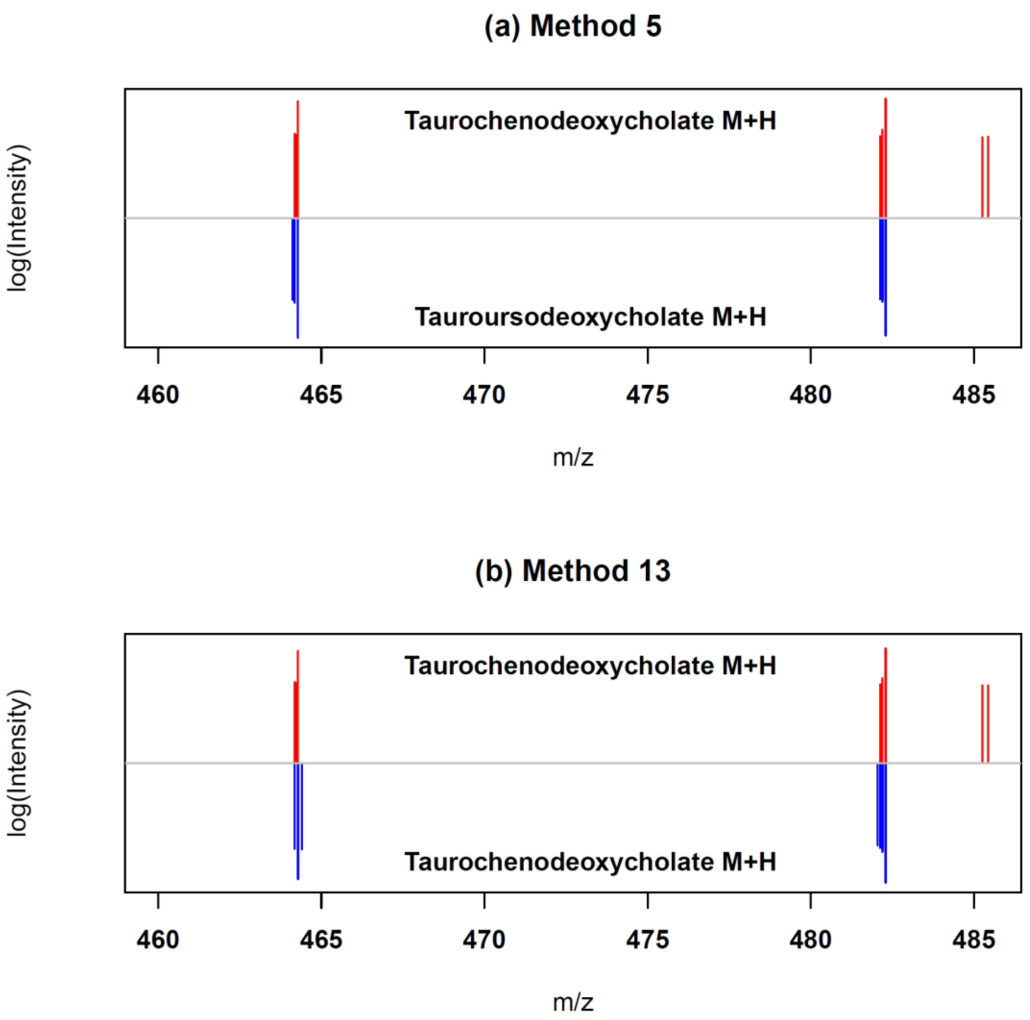

Figure S6. A case that Method 5 (Cosine) matches incorrectly while Method 13 (Intersection) matches correctly for ESI, where the m/z range for Figure S5 is shorten. The red bars represent the query spectrum and the blue bars the library spectrum with the highest similarity score correponding to the binary similarity measure.

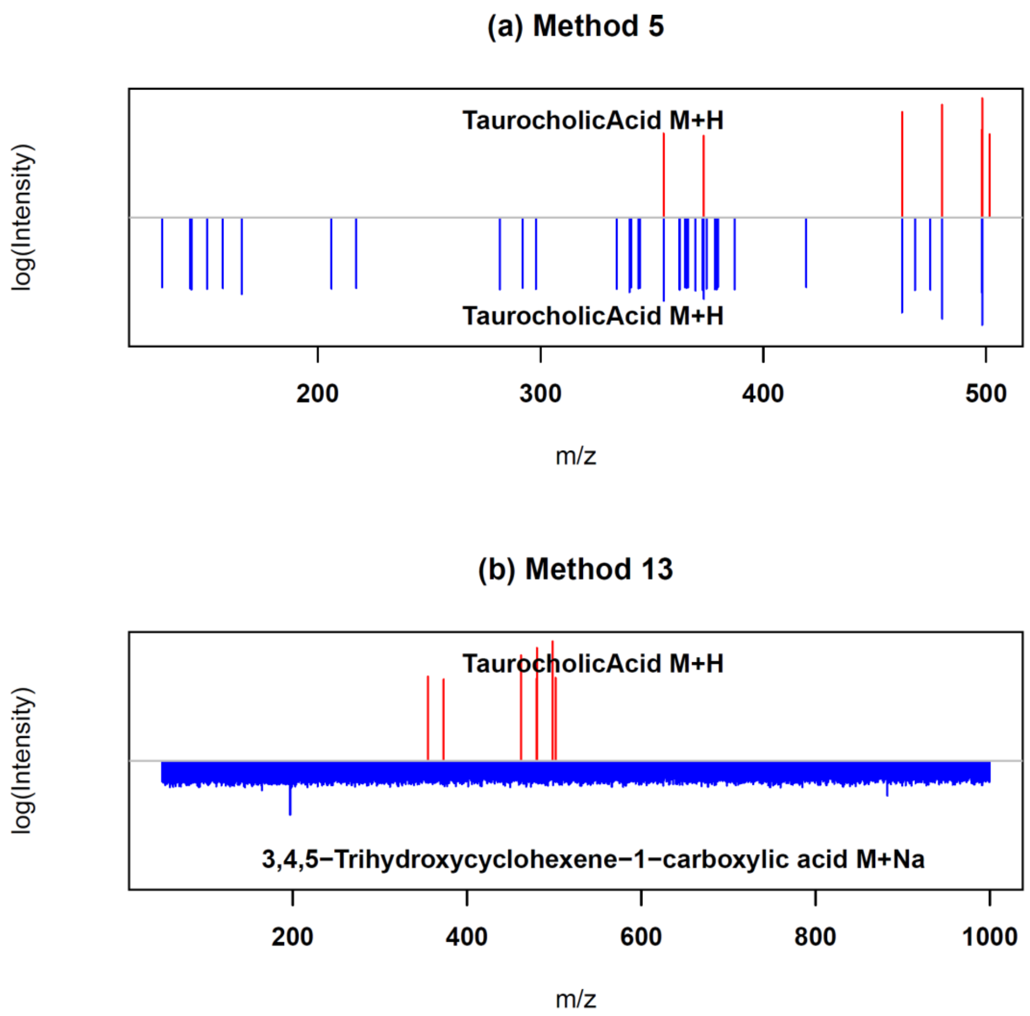

Figure S7. A case that Method 5 (Cosine) matches correctly while Method 13 (Intersection) matches incorrectly for ESI. The red bars represent the query spectrum and the blue bars the library spectrum with the highest similarity score correponding to the binary similarity measure.

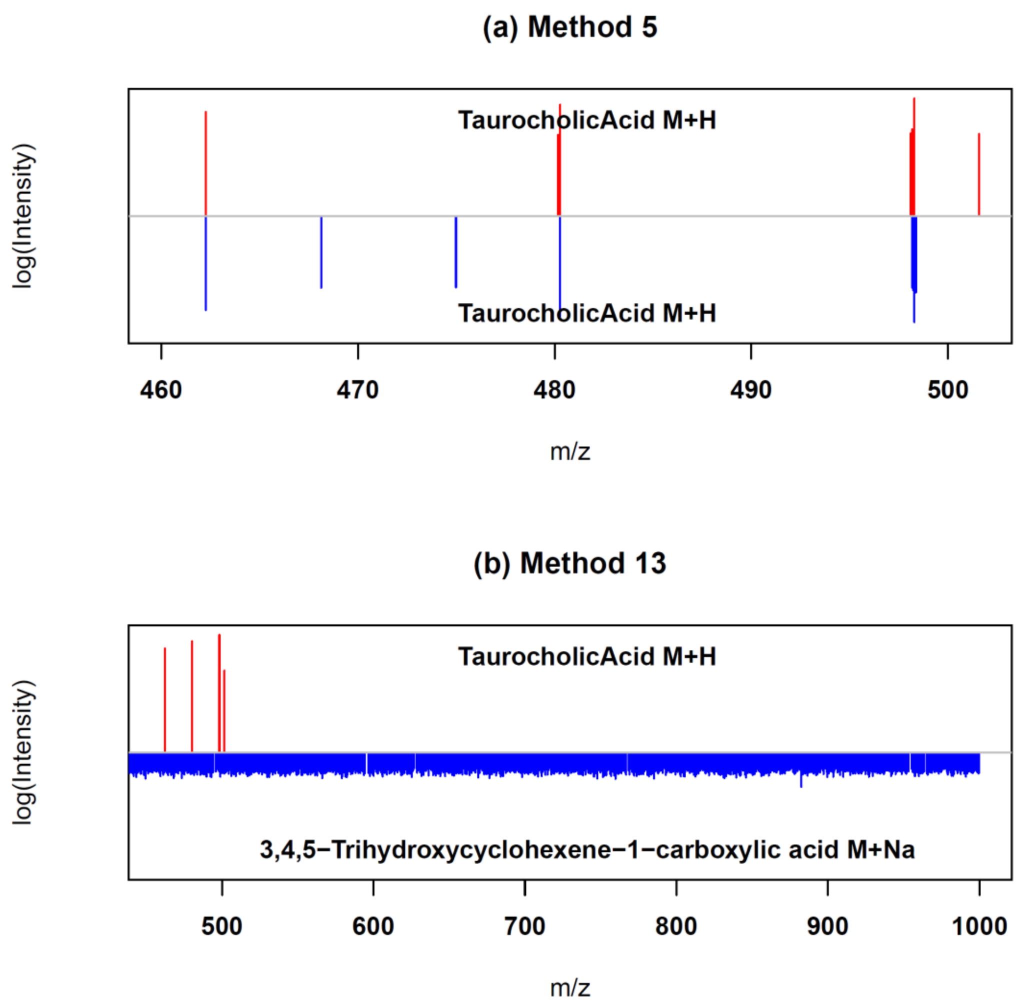

Figure S8. A case that Method 5 (Cosine) matches correctly while Method 13 (Intersection) matches incorrectly for ESI, where the m/z range for Figure S7 is shorten. The red bars represent the query spectrum and the blue bars the library spectrum with the highest similarity score correponding to the binary similarity measure.
